# Volunteering and Chronic Inflammation in Later Life: Is Sustained Volunteering Beneficial for Health?

**DOI:** 10.1093/geroni/igab046.2594

**Published:** 2021-12-17

**Authors:** Mallory Bell, Madison Sauerteig, Kenneth Ferraro

**Affiliations:** 1 Purdue, West Lafayette, Indiana, United States; 2 Purdue University, West Lafayette, Indiana, United States

## Abstract

Although research on the health benefits of volunteering has proliferated in recent decades, most studies have focused on whether or not a person volunteers or the monthly frequency of volunteering. This study examines whether sustained volunteering has health benefits above and beyond occasional or short-lived volunteering. To investigate the salubrious effects of volunteering, the present study considers sustained volunteering engagement in terms of both formal and informal volunteering. Using four waves of data from the Health and Retirement Study, we assess the influence of sustained volunteering on chronic inflammation, measured by C-reactive protein (CRP). Results reveal that sustained engagement in formal and informal volunteering is related to lower CRP concentration, but this association is partly mediated by adult health and socioeconomic factors. Although sustained volunteering is associated with lower levels of chronic inflammation, older adults who maintain their volunteering over time are a select category of adults, characterized by higher education and wealth and better health.

